# Focal precentral gyrus involvement in osmotic demyelination

**DOI:** 10.1136/practneurol-2017-001682

**Published:** 2017-08-19

**Authors:** Martin R Turner, John Jacob, Lucy Matthews, Fintan Sheerin

**Affiliations:** 1 Nuffield Department of Clinical Neurosciences, Oxford University, John Radcliffe Hospital, Oxford, UK; 2 Department of Neurology, Milton Keynes University Hospital, Milton Keynes, UK; 3 Department of Neurology, John Radcliffe Hospital, Oxford University Hospitals NHS Foundation Trust, Oxford, Oxfordshire, UK

**Keywords:** motor neuron disease, biochemistry, metabolic disease, neuroanatomy, magnetic resonance imaging

## The case

**Figure 1:**
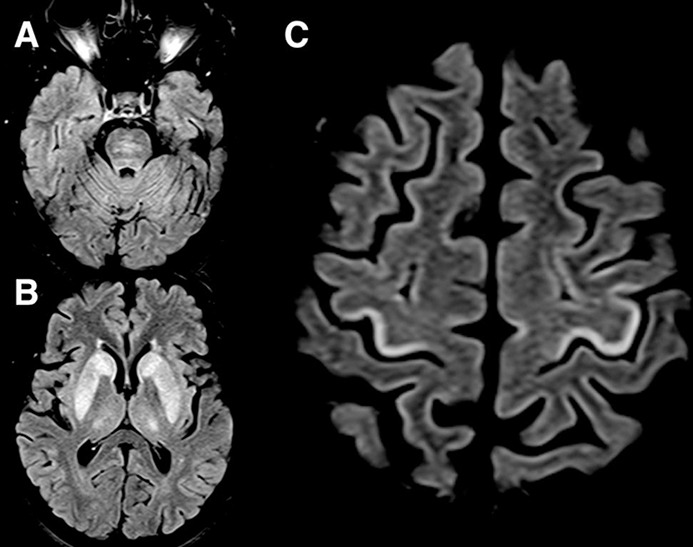
MR scan of the brain. Axial fluid-attenuated inversion recovery (FLAIR) images show symmetrical pontine (A), basal ganglia (B) and a ‘ribbon’ of cortical T2 hyperintensity in the posterior precentral gyri (C). The precentral gyral changes are strikingly similar to a focal pattern of MRI changes described in cases of amyotrophic lateral sclerosis.

A previously well 59-year-old woman was admitted to a regional hospital with subacute encephalopathy and found to have severe hyponatraemia (101 mmol/L). She had taken indapamide for hypertension over the preceding 3 months. Before referral to neurological services, she had received intravenous hypertonic saline. Her serum sodium concentration normalised within a few days, but was associated with a deteriorating conscious level. She was referred to our tertiary neurological centre with suspected osmotic demyelination where she presented in a ‘locked in’ state, involving quadriplegia and absent vertical eye movements. MR scan of the brain showed typical symmetrical pontine and basal ganglia changes, but also focal hyperintensity of the precentral gyri (figure 1). Nearly 6 months after the initial admission to hospital, the patient had made some recovery of limb strength, but with persistent loss of fine dexterity and unable to stand independently.

Hyponatraemia secondary to thiazide diuretics is well recognised, and cautious correction of chronic hyponatraemia is generally advised to minimise the risk of osmotic demyelination. This unusual extension of MRI changes in extrapontine myelinolysis is uncommon,^1^ and our case also shared the bilateral hyperintensity in the posterolateral nuclei of the thalami. The strikingly selective involvement of the posterior border of the precentral gyrus is more commonly described in cases of amyotrophic lateral sclerosis in relation to upper motor neurone degeneration,^2 3^ where iron deposition has been inferred. Corticospinal tract MRI white matter changes occurring in patients with fulminant hepatic failure also overlap in their appearance with those seen in cases of amyotrophic lateral sclerosis.^4^ These observations might reflect a hitherto unrecognised selective anatomical connectivity of subcortical structures with this region of the motor cortex, or a shared focal metabolic susceptibility.
